# Local anesthetic injections with or without steroid for chronic non-cancer pain: a protocol for a systematic review and meta-analysis of randomized controlled trials

**DOI:** 10.1186/s13643-016-0190-z

**Published:** 2016-02-01

**Authors:** Harsha Shanthanna, Jason W. Busse, Lehana Thabane, James Paul, Rachel Couban, Harman Choudhary, Alka Kaushal, Erica Suzumura, Isabel Kim, Prathiba Harsha

**Affiliations:** Department of Anesthesia, McMaster University, St. Joseph’s Healthcare Hamilton, 50 Charlton Avenue East, Hamilton, ON L8N 4A6 Canada; The Michael G. DeGroote Institute for Pain Research and Care, Hamilton, Canada; Department of Clinical Epidemiology and Biostatistics, McMaster University, Hamilton, Canada; Department of Orthopedics, McMaster University, Hamilton, Canada; Research Institute - Hospital do Coração (HCor), São Paulo, Brazil; Michael G. DeGroote School of Medicine, McMaster University, Ontario, Hamilton Canada

**Keywords:** Chronic non-cancer pain, Steroid injections, Nerve blocks, Epidurals, Intra-articular, Local anesthetic, Systematic review, Meta-analysis

## Abstract

**Background:**

Steroids are often combined with local anesthetic (LA) and injected to reduce pain associated with various chronic non-cancer pain (CNCP) complaints. The biological rationale behind injection of a steroid solution is unclear, and it is uncertain whether the addition of steroids offers any additional benefits over injection of LA alone. We propose to conduct a systematic review and meta-analysis to summarize the evidence for using steroids and LA vs. LA alone in the treatment of CNCP.

**Methods:**

An experienced librarian will perform a comprehensive search of EMBASE, MEDLINE, and the Cochrane Central Registry of Controlled Trials (CENTRAL) databases with search terms for clinical indications, LA, and steroid agents. We will review bibliographies of all relevant published reviews in the last 5 years for additional studies. Eligible trials will be published in English and randomly allocate patients with CNCP to treatment with steroid and LA injection therapy or injection with LA alone. We will use the guidelines published by the Initiative on Methods, Measurement, and Pain Assessment in Clinical Trials (IMMPACT) to inform the outcomes that we collect and present. Teams of reviewers will independently and in duplicate assess trial eligibility, abstract data, and assess risk of bias among eligible trials. We will prioritize intention to treat analysis and, when possible, pool outcomes across trials using random effects models. We will report our findings as risk differences, weighted mean differences, or standardized mean differences for individual outcomes. Further, to ensure interpretability of our results, we will present risk differences and measures of relative effect for pain reduction based on anchor-based minimally important clinical differences. We will conduct a priori defined subgroup analyses and use the Grading of Recommendations Assessment, Development, and Evaluation (GRADE) system to evaluate the certainty of the evidence on an outcome-by-outcome basis.

**Discussion:**

Our review will evaluate both the effectiveness and the adverse events associated with steroid plus LA vs. LA alone for CNCP, evaluate the quality of the evidence using the GRADE approach, and prioritize patient-important outcomes guided by IMMPACT recommendations. Our results will facilitate evidence-based management of patients with chronic non-cancer pain and identify key areas for future research.

**Trial registration:**

PROSPERO CRD42015020614

**Electronic supplementary material:**

The online version of this article (doi:10.1186/s13643-016-0190-z) contains supplementary material, which is available to authorized users.

## Background

### Burden of the problem

Chronic non-cancer pain (CNCP), defined as pain persisting beyond the period of normal healing (typically ≥3 months), is commonly experienced by a large proportion of adult population. In the USA, according to 2008 estimates, approximately 100 million adults were affected by CNCP with associated treatment costs ranging from $560 to $635 billion (US). This is greater than annual treatment costs of heart disease, cancer, or diabetes [1]. In the UK, 53 % of seniors reported that CNCP was the most important factor impacting their quality of life [2]. Chronic low back pain (CLBP) has been noted to be the leading cause of years lived with disability, with neck pain as the fourth common cause [3]. CNCP accounts for a large number of physician encounters in the elderly and aging population. In the USA alone, the proportion of CLBP patients requesting care from a health provider increased from 73.1 to 84.0 %, between 1992 and 2006 [4]. Conditions characterized or defined by the presence of pain accounted for five of the leading ten conditions associated with the most years lived with disability [5]. Common peripheral (non-axial) CNCP conditions include pain in upper or lower limbs, knee and shoulder joints, headache, carpal tunnel syndrome, epicondylitis, fascitis and others.

### Treatment considerations

A clear understanding of the underlying pathogenesis of CNCP is important to drive treatment decisions; however, despite decades of research, the mechanisms underlying most CNCP conditions are unclear [5]. CNCP conditions involve a complex interplay of active pain pathways leading to sensory signaling, along with peripheral and central sensitization as a result of nervous system changes induced by temporary and long-term neuroplasticity. Changes resulting from sensitization lead to modulation of pain signals, which vary from patient to patient. Most CNCP conditions do not have definitive treatment, and physicians have limited options for symptomatic management. Analgesic medications are not always effective and need to be given on a daily basis; they also involve a risk of drug interaction especially in the elderly adult population. Corticosteroid injections (CSI) are commonly used to manage many CNCP conditions, despite variability in their clinical presentation and underlying pathophysiology. For neuraxial pain, epidural steroid injections (ESI) are the most commonly performed interventions, and in the USA alone, the number of ESIs administered to Medicare recipients essentially doubled between 2000 and 2004 (from 740,845 to 1,437,962 procedures/year) [6]. CSIs are also widely used as injections to knee [7], shoulder [8], carpal tunnel [9], nerve blocks for occipital nerve [10], suprascapular nerve [11], tendinous injections for medial and lateral epicondyle [12], and even for simple trigger point injections [13].

### Percutaneous injections using steroids and local anesthetics

The rationale for injecting local anesthetics (LA) is to block sensory signals from the region being injected. Although often used for diagnosis—because it should only lead to a temporary blockade—an injection of LA has the potential to decrease sensitization, which is a feature of chronic or persistent pain, thereby possibly prolonging the treatment effect of LA beyond its pharmacological duration of action [14]. Historically, many investigators have published on the successful use of epidural injections of saline and LA. Cathelin. F, Pasquier and Leri, and Sicard published reports as early as 1901 [15]. Later, Evans (1931) published a successful report using procaine and saline in 22 of 40 patients [16]. Cyriax published multiple manuscripts referring to safe use of epidural injections using only LA and steroids in more than 20,000 cases [17, 18]. In fact, up until the 1950s, the injectate used for epidural injections in sciatica consisted of LA and saline. The first recorded use of steroids in epidural space was by Lievre et al. in 1953 [19]. Discovered by Philip Hench in the 1940s [20], glucocorticoids are potent anti-inflammatory agents. Since then, steroids have been injected for nearly every chronic pain indication [21]. In clinical practice, steroids are typically combined with LA, with or without saline [15]. This practice stems from the hope that the addition of steroid can lengthen the treatment effect [22]. For most CNCP conditions (except for evidently inflammatory conditions such as rheumatoid arthritis), there is no evidence that CSI are disease-modifying agents [23]. Whether steroids have any direct effect on pain generation or transmission is not clear. There is some experimental evidence demonstrating suppression of ectopic discharge in neuromas [24]. Pre-clinical experiments suggest that steroids may reduce neuropathic pain; however, a paradoxical effect of increased pain in some patients has also been shown [25]. Surprisingly, there are few clinical studies that have attempted to elicit the mechanism behind steroid’s effect on chronic pain other than as an anti-inflammatory [26]. Thus, the use of steroid for interventions in CNCP lacks clear rationale.

### Harms of steroid injections

Corticosteroids can have systemic and local adverse effects. Systemic side effects are largely dependent upon the patient’s physiology, injected dose, and systemic absorption. Theoretically, these systemic effects can occur when the dose of steroids injected exceeds the rate of endogenous steroid production of about 20 mg per day of hydrocortisone or its equivalent and potentially cause suppression of pituitary adrenal axis, hypercorticism, Cushing’s syndrome, osteoporosis, avascular necrosis of bone, steroid myopathy, weight gain, fluid retention, and hyperglycemia [21]. Although these risks are unlikely with a single injection, in clinical practice most patients receive multiple CSI injections at frequent intervals, and it is not uncommon for patients to suffer from multiple chronic pain conditions treated with steroid injections by different physicians, without necessarily accounting for the systemic effects of steroid injected elsewhere. Local adverse effects include soft tissue atrophy, depigmentation [27], and alopecia [28]. Beyond the above recognized adverse effects, the neuraxial steroid injections (epidural, facet joint) risk causing rare, but catastrophic neurologic injuries such as stroke and spinal cord injury [29].

### Limitations of previous reviews

Existing systematic reviews of injection therapy for CNCP conditions have not specifically compared LA vs. LA and steroid injections. Further, some reviews have considered LA injection to be a neutral agent, and combined this intervention with other biologically inactive control agents such as saline injections [12, 30]. More recently, within the last quarter of 2015, two additional reviews have been published. Manchikanti et al. reviewed all spinal injections targeted at epidural, facet joint/nerve, and disc pain conditions. Due to the existing heterogeneity, a meta-analysis was not considered feasible. All RCTs using an active control design were included. They observed that LA alone was as equally effective as LA with steroid, except in disc herniations [18]. The review by Chou et al. also focussed only on epidural injections for radiculopathy and spinal stenosis, without any restriction for the agent used and duration of chronic pain. They combined LA along with saline as placebo comparator. Their results showed a small effect favoring the use of steroids only in radiculopathy for short-term reduction in pain and function [31]. To illustrate these issues, we reviewed the systematic reviews published in English within the last 5 years, focusing on injection therapies using CSI for CNCP conditions. We identified the findings and limitations of those reviews, apart from identifying whether they included trials that addressed our review question of comparing LA and steroid with LA alone for CNCP (Table 1). Twenty seven out of forty-two reviews included trials comparing LA and steroid with LA alone [6, 12, 18, 30–53], but only some reviews have made clear observations on the comparison between LA vs. LA and steroid [18, 33, 38, 41, 45, 48, 51]. Very few conducted meta-analyses [12, 31–33, 35, 39, 41, 45, 48, 51]. The question of whether the injected steroid has any clinical benefit beyond that achieved from LA alone is an area of active debate, as existing studies across various clinical categories have shown equivalent effects comparing LA and steroid vs. only LA [54–57]. Findings from our review will help inform physicians, healthcare providers, and CNCP patients on whether the addition of steroid gives any meaningful benefit to LA injection alone.

**Table 1:** Limitations of existing reviews identified by a systematic search of literature

Serial No	Main Objective	Relevant Findings and Limitations	Studies relevant to our Review
1	A systematic review and meta-analysis of RCTs evaluating the "control" injections in epidural injections for spinal pain [35].	FINDINGS: As control injections, epidural non-steroid injections may provide some benefit, but were inferior to ESI, but superior to non-epidural injections.	Anderberg 2007 [80]; Beliveau 1971[81]; Brevik 1996 [82]; Cohen 2012 [83]; Cuckler 1985 [84]; Ghahreman 2010 [85]; Klenerman 1984 [86]; Manchikanti 2008 [87], 2011 [88], 2012abcd [89–92]; Nam & Park 2011 [93]; Ng 2005 [94]; Rogers 1992 [95]; Sayegh 2009 [96]; Tafazal 2009 [97]
		LIMITATIONS: All LA and saline comparators were grouped as epidural non-steroid agents.	
2	To assess comparative effectiveness studies in ESI for Lumbar Spinal Stenosis and to estimate reimbursement amounts [37].	FINDINGS: Both, ESIs or LA epidural injections alone, resulted in better short term improvement (pain and walking distance); no longer term difference.	Fukusaki-1998 [98]; Cuckler 1985 [84]; El Zahaar 1991 [99]
		LIMITATIONS: Included both RCTs and OSs; no metaanalysis.	
3	Effectiveness of cervical epidural injections in the management of chronic neck and upper extremity pain [38].	FINDINGS: Similar effectiveness with both LA only and LA+ steroid injections, except for slightly better results with radiculitis from disc herniations	4 by Manchikanti: 2010 [100]; 2012a; 2012b; 2012c [101–103]
		LIMITATIONS: Included RCTs had differences in the injectate used with intervention and control arms; no metaanalysis.	
5	Effectiveness and risks of image guided cervical TFESI [30].	FINDINGS: Limited evidence exists and no conclusion on effectiveness and risks can be observed.	Anderberg 2007 [80]
		LIMITATIONS: Included three RCTs, only one of which compared LA+ steroid with LA only	
6	Role of ESIs in the prevention of surgery for spinal pain [36].	FINDINGS: ESIs may provide a small surgery-sparing effect in the short term compared with control injections.	Hegihara 2008 [104]; Klenerman 1984 [86]; Cohen 2012 [83]; Cuckler 1985 [84]; Ghahreman 2010 [85]; Riew 2000 [105]; Sayegh 2009 [96]; Tafazal 2009 [97];
		LIMITATIONS: Looked only at surgery sparing effects; no metaanalysis	
7	ESIs in the management of sciatica [6].	FINDINGS: Small short term benefit in pain control with ESIs.	Manchikanti 2010a,b; Ghahreman 2010 [85]; Tafazal 2009 [97]; Ng 2005 [94]; Rogers 1992 [95]; Cuckler 1985 [84]; Klenerman 1984 [86]; Swerdlow 1970 [106]
		LIMITATIONS: No differentiation was made with the injectate used in control and treatment arm. Could not incorporate dichotomous outcome measures into pooling.	
8	The effectiveness of lumbar interlaminar ESIs in managing chronic low back and lower extremity pain [33].	FINDINGS: Similar results with both LA only and LA+ steroid injections, except for slightly better results with radiculitis from disc herniations.	Manchikanti 2010a,b [107, 108]; Cuckler 1985; Rogers 1992
		LIMITATIONS: Included both RCTs and OSs without any pooling.	
9	Predicting ESIs with lab markers and imaging techniques [68].	LIMITATIONS: Only aimed at prognostic accuracy of certain predictive methods used to determine ESI outcomes.	None
10	A systematic evaluation of thoracic ESIs [34].	FINDING: The single RCT showed similar effectiveness with LA or LA +steroid.	Manchikanti. 2010 [109]
		LIMITATIONS: Only one RCT, and one OS were included	
11	Effectiveness of TFESI for lumbar radiculopathy [39].	FINDINGS: Small improvement with steroids in pain only (short term); long term follow up showed no difference with steroids.	Riew 2000 [105]; Ng 2005 [94]; Tafazal 2009 [97]
		LIMITATIONS: Included only five RCTs, and for pooling control groups included both LA and Saline; outcomes as SMD	
12	Evaluation of therapeutic lumbar TFESIs [42].	FINDINGS: Lack of evidence	Riew 2000 [105]; Riew 2006 [110]
		LIMITATIONS: Only four RCTs; no metaanalysis; comparators varied in each study	
13	Efficacy of lumbosacral TFESIs: a systematic review [49].	FINDINGS: Fair evidence supporting TFESIs as superior to placebo for treating radicular symptoms.	Riew 2000 [105]; Ng 2005 [94]
		LIMITATIONS: Evaluation specific to TFESI; no metaanalysis; varied comparators.	
14	Evaluation of perineural steroids for trauma and compression-related peripheral neuropathic pain [41].	FINDINGS: At 1–3 months post-interventions, steroid group reported lower pain scores than those who received LA or conventional care.	Karakadas 2011, 2012 [111, 112]; Eker 2012 [113]; Thomson 2013 [114]
		LIMITATIONS: Review limited to compression neuropathies; comparators for pooling included no injection, or LA, or placebo (saline).	
16	Evaluation of PNBs and TPIs in headache [40].	FINDINGS: Lack of studies and inherent limitations within the included studies.	Ashkenazi 2008 [115]
		LIMITATIONS: Did not identify any study on TPI; both RCTs and non-RCTS were included; no assessment of risk; no metaanalysis.	
17	Treatment of carpal tunnel syndrome [43].	FINDINGS: Local steroid injection is recommended before surgery.	Armstrong 2004 [116]
		LIMITATIONS: A report as guidelines for management based on previous systematic reviews; however no differentiation between steroids with or without LA.	
18	Neural blockade for persistent pain after breast cancer surgery [69].	FINDINGS: Lack of evidence.	None
		LIMITATIONS: Only two RCTs on stellate ganglion block.	
19	Occipital nerve blocks: when and what to inject [52].	LIMITATIONS: Narrative review with search obtained from google scholar and MD consult	Afridi 2006 [117]; Ambrosini 2005 [118]; Ashkenazi 2008 [115]
20	IA infiltration therapy for patients with glenohumeral osteoarthritis [70].	FINDINGS: No clear conclusions on the use of IA steroid due to lack of evidence.	None
		LIMITATIONS: Studies of all kinds of injection treatments; only two RCTs of IA injection involving hyaluronic acid.	
21	A metaanalysis of steroid injections for painful shoulder [32].	FINDINGS: Subacromial injections of steroids are effective for improvement for rotator cuff tendonitis, and are better than NSAIDS and placebo injections.	Blair 1996 [119]; Plafki 2000 [120]; Vecchio 1993 [121]
		LIMITATIONS: Out of five RCTs included for pooling only three compared LA + steroid vs LA; results not considered separately.	
22	Review of glenohumeral steroid injections in adhesive capsulitis [71].	FINDINGS: Steroids injections offer good short-term outcomes when compared to physical therapy and other treatments.	None
		LIMITATIONS: Although 16 RCTs were included, none of them compared LA + steroid with only LA.	
23	Assessment of Subacromial steroid injections in the treatment of rotator cuff disease [44].	FINDINGS: Little reproducible evidence to support the efficacy of subacromial steroid injections in managing rotator cuff disease.	Akgun 2004 [122]; Alvarez 2005 [123]; Blair 1996 [119]; Petri 1987 [124]; Withrington 1985 [125]
		LIMITATIONS: Out of nine RCTs, three involved patients with acute pain; no metaanalysis; varying comparators within the studies.	
24	IA cortisone injection for osteoarthritis of the hip. Is it effective and safe [46]?	FINDINGS: Lack of clear evidence; steroid injections are better in refractory pain; of the four RCTs- two of the trials showed opposite results with LA vs LA + steroid	Lambert 2007 [126]; Flanagan 1988 [128]
		LIMITATIONS: Identified only four RCTs; no metaanalysis.	
25	Is anesthetic Hip Joint Injection Useful in Diagnosing Hip Osteoarthritis? A Meta-Analysis of Case Series [72].	LIMITATIONS: Only non-RCTs, and does not allow for clear conclusions or directions.	None
26	Injection therapies in LE: a systematic review and network meta-analysis of RCTs [45].	FINDINGS: No statistically significant difference in benefit compared with placebo for steroid injections. LIMITATIONS: Network meta-analysis involving 10 trials of steroid injections; LA was not considered as a separate comparison group vs LA+ steroid.	Dogramaci 2009 [128]; Lindenhovious 2008 [129]; Newcomer 2001 [130]; Price 1991a,b [131, 132]
27	Treating LE with steroid injections or physiotherapy: a systematic review [48].	FINDINGS: For steroid vs LA injection, the evidence is conflicting; steroid injections have a short term beneficial effect, but a negative effect in the intermediate term.	Lindenhovious 2008 [129]; Newcomer 2001 [130]; Price 1991 [131]
		LIMITATIONS: Outcomes pooled separately, and expressed as SMD for continuous and RD for dichotomous	
28	To assess the effectiveness of interventions for cubital tunnel syndrome, radial tunnel syndrome, instability, or bursitis of the elbow: a systematic review [73].	FINDINGS: No or limited evidence found for the effectiveness of nonsurgical and surgical interventions; lack of good controlled studies.	None
		LIMITATIONS: Various interventions with varying comparators; no studies relevant to LA vs LA +steroid; no metaanalysis.	
30	To evaluate the effectiveness of corticosteroid injections for lateral epicondylitis [51].	FINDINGS: For studies (3) comparing LA vs steroid, beneficial effects were found favoring steroid injections.	Price 1991 [131]
		LIMITATIONS: Out of 15 RCTs, five compared LA with LA and steroid. Outcomes with various comparators pooled together.	
31	Non-surgical treatment of LE: a systematic review of RCTs [50].	FINDINGS: Existing literature does not provide conclusive evidence for a preferred mode of nonsurgical treatment.	Lindenhovious 2008 [129]; Newcomer 2001 [130]; Dogramaci 2009 [128]; Altay 2002 [130]
		LIMITATIONS: Various non-surgical treatments were considered together; no metaanalysis	
32	Assessing the efficacy and safety of steroid injections and other injections for management of tendinopathy [12].	FINDINGS: For LE: Steroid injections reduced pain in the short term; but studies comparing only LA showed conflicting results; rotator tendinopathy results are conflicting; Achilles and Patellar tendinopathies-no studies of comparison; ME-no benefit from steroid injection.	LE: Lindenhovious 2008 [129]; Newcomer 2001 [130]; Price 1991 [131]
			ME: Stahl 1997 [134]
		LIMITATIONS: The effect of steroid injections were compared using all comparators; no separate analysis with LA + steroid vs only LA.	RT: Adebajo 1990 [135]; Alvarez 2005 [123]; Blair 1996 [119]; Ekeberg 2009 [136]; Mclnerney 2003 [137]
33	Evaluation of minimally invasive therapies in the management of chronic calcific tendinopathy of the rotator cuff. [74].	FINDINGS: Lack of evidence.	None
		LIMITATIONS: Did not identify any studies comparing steroid injection with LA.	
34	Efficacy of treatment of trochanteric bursitis: a systematic review [75].	FINDINGS: Lack of evidence.	None
		LIMITATIONS: Only one RCT for steroid injection assessing image guidance.	
35	Evaluation of non-operative management of discogenic back pain [76].	FINDINGS: Lack of evidence.	None
		LIMITATIONS: Identified only two RCTs performing intradiscal steroid injections; no study compared LA + steroid vs LA	
36	Evaluation of various modes of diagnosis and treatment of suspected discogenic pain [77].	FINDINGS: There is lack of diagnostic criteria and lack of studies with uniform treatment strategies.	None
		LIMITATIONS: Did not identify any suitable RCTs.	
37	Evaluation of therapeutic thoracic facet joint interventions [47].	FINDINGS: Paucity of evidence, but one trial showed no difference between LA+ steroid vs LA.	Manchikanti 2012 [138]
		LIMITATIONS: Identified only one RCT on nerve block; no study on joint injections	
38	Effectiveness of therapeutic lumbar facet joint interventions [53].	FINDINGS: Paucity of evidence, but one trial showed no difference between LA+ steroid vs LA.	Manchikanti 2001 [139]
		LIMITATIONS: Identified only one RCT on nerve block; no study on joint injections.	
39	Emerging concepts in the treatment of myofascial pain: a review of medications, modalities, and needle-based interventions [78].	FINDINGS: There is insufficient evidence for both medications and needle based interventions for myofascial pain.	None
		LIMITATIONS: Did not identify any RCT comparing LA + steroid vs LA.	
40	To assess the efficacy and safety of using TPI to treat patients with chronic non-malignant musculoskeletal pain [79].	FINDINGS: No clear evidence to support the use of TPI.	None
		LIMITATIONS: Did not identify any RCT comparing LA + steroid vs LA.	
41	To compare the efficacy of saline, LA, and steroids in epidural and facet joint injections for the management of spinal pain [18].	FINDINGS: LA with steroids and LA alone were equally effective except in disc herniation, where the superiority of LA with steroids was demonstrated over LA alone.	Anderberg 2007 [80]; Beliveau 1971 [81]; Brevik 1996 [82]; Cohen 2012 [83]; Cuckler 1985 [84]; Ghahreman 2010 [85]; Klenerman 1984 [86]; Manchikanti 2008 [87], 2011 [88], 2012abcd [89–92]; Nam & Park 2011 [93]; Ng 2005 [94]; Rogers 1992 [95]; Sayegh 2009 [96]; Tafazal 2009 [97]
		LIMITATIONS: RCTs involving the injections of sodium chloride solution was also included as active comparator, along with LA alone injections.	
		Studies were not excluded based on the duration of chronic pain.	
		No metaanalysis was done.	
42	To assess the benefits and harms of ESIs in adults with radicular low back pain or spinal stenosis of any duration [31].	FINDINGS: For radiculopathy, small effect favoring the use of steroids for short term reduction in pain and function. No evidence of benefit in spinal stenosis.	Anderberg 2007 [80]; Beliveau 1971 [81]; Brevik 1996 [82]; Cohen 2012 [83]; Cuckler 1985 [84]; Ghahreman 2010 [85]; Klenerman 1984 [86]
		LIMTATIONS: Combined all non-steroid agents as placebo comparators.	
		Focused on radicular pain, but included studies of any duration.	

*Abbreviations*: *LA* local anesthetic; *RCT* randomized control trial; *OS* observational study; *ESI* epidural steroid injection; *TFESI* transforaminal epidural steroid injection; *SMD* standard mean deviation; *PNB* peripheral nerve block; *TPI* trigger point injection; *LE* lateral epicondylitis; *ME* medial epicondylitis; *RD* risk difference; *IA* intra-articular injection

## Objectives

Our primary objective is to perform a systematic review and meta-analysis to assess the effectiveness of steroid + LA injections compared to LA injections, for pain relief among CNCP patients. Secondary objectives of our review are

- To assess the effect of steroid + LA injections compared to LA injections alone on the duration of effect of pain relief.
- To assess the effect of steroid + LA injections compared to LA injections alone on six core domains, described as core outcome measures by the Initiative on Methods, Measurement, and Pain Assessment in Clinical Trials (IMMPACT) [58]. The domains other than reduction of pain include physical functioning; emotional functioning; participant ratings of global improvement and satisfaction with treatment, symptoms and adverse effects, and participant disposition.

### Study question

Among adults with CNCP, does LA with corticosteroid injection (with or without saline) offer better pain relief when compared to injection with LA alone (with or without saline), as assessed at a time closest to 4 weeks after treatment?

## Methods

Our review protocol has been registered with PROSPERO (registration number PROSPERO 2015:CRD42015020614). This protocol has been prepared for publication according to PRISMA-P guidelines [59].

### Eligibility criteria

#### Participants

We will include adult (≥18 years of age) patients with CNCP, and exclude patients with a known inflammatory cause of pain such as rheumatoid arthritis, ankylosing spondylitis, systemic lupus erythematosus and gouty arthritis, and pain of generalized nature such as fibromyalgia or chronic fatigue syndrome. If a trial involves a mix of cancer and CNCP, or adult and pediatric patients, we will include the study only if they report outcomes separately for our study population of interest, or if at least 90 % of the trial patients are >18 years with CNCP.

### Studies

Parallel design, randomized controlled trials (RCTs) will be eligible for our review. We will exclude trials with crossover design, *N* of 1 trials, and non-therapeutic trials.

### Interventions

Eligible studies must randomize patients to receive LA + steroid or LA only, with or without saline, administered as percutaneous injection. We will exclude trials in which injections involve any additional agent (e.g., Hyaluronidase, dextrose, plasma), and any injection which involves a co-interventional procedure (e.g., radiofrequency treatment, epidurolysis).

### Information sources

We will search the following electronic databases, from its inception till our search date: EMBASE, MEDLINE, and the Cochrane Central Registry of Controlled Trials (CENTRAL). Our search will not be limited to language. We will record the time and date of the literature search performed on each database. As a supplementary search, we will search the WHO clinical trial registry (http://apps.who.int/trialsearch/Default.aspx), and clinical trial registry (https://clinicaltrials.gov/), to look for any registered studies, which fulfill our eligibility criteria, and crosscheck for published results. Unpublished, but completed, study results will be requested from the authors or investigators. To further ensure comprehensiveness, we will review the bibliographies of relevant reviews published in English over the last 5 years (shown in Table 1).

### Search strategy

The search will be performed using a sensitive strategy, prepared by an experienced librarian (RC), for each specific database, based in part on a comprehensive list of CNCP indications for which CSI are commonly utilized. The search terms will include possible indications, along with terms identifying corticosteroids, and LAs (Additional file 1).

### Study records

#### Data management

We will conduct our review using an online software tool specially optimized to conduct systematic reviews—DistillerSR (https://distillercer.com/).

### Study screening and selection

Studies fulfilling our eligibility criteria will be selected through a two-level screening process using standardized forms, applied through DistillerSR. At each stage, paired reviewers trained in health research methods will screen for studies for eligibility, independently and in duplicate. The first level will be done on titles and available abstracts of identified citations. For citations judged as potentially eligible, full-text article screening will be done. To ensure consistency, reviewers will perform a calibration exercise, before beginning with screening. Reviewers will be asked to resolve disagreement by consensus, or if a discrepancy remains, through discussion with an arbitrator (HS). A quadratic kappa statistic will be calculated as a measure of inter-observer agreement, independent of chance regarding study eligibility and interpreted as almost perfect agreement (0.81–0.99), substantial agreement (0.61–0.80), moderate agreement (0.41–0.60), fair agreement (0.21–0.40), slight agreement (0.01–0.20), <0 as less than chance agreement [60].

### Data collection process

Reviewers working in pairs will extract the required data from each included study using a standardized data extraction form using DistillerSR (https://distillercer.com/). This form will be piloted between all pairs of reviewers for consistency and accuracy. To assist with the data extraction, a detailed instruction manual will be provided along with each relevant form.

### Data items

Data abstracted will include study characteristics including risk of bias items, demographic information, participant flow through the study, and outcomes on continuous and binary measures captured on six core domains as recommended by the IMMPACT statement guidelines [58].

### Outcomes and prioritization

We will consider pain relief as our primary outcome. We will also capture other outcomes (as guided by IMMPACT) including reporting of adverse effects. We will also prioritize the use of intention to treat analysis (ITT). We will only pool data across trials if there are three or more studies contributing to an outcome domain. It is recognized that presentation of relative effects will facilitate interpretation of treatment effects, and clinicians generally find dichotomous presentation of continuous outcomes more useful [61]. In the following section, we will explain the method of analysis as considered for pain relief. We will perform a similar analysis, as appropriate, for other IMMPACT outcomes.

### Data synthesis and analysis of outcomes

We expect that all included trials will have captured a measure of pain relief, and this domain could be expressed in any of the following types: outcomes reported as binary (successful or not successful), outcomes reported in ordinal categorical scale (mild, moderate and severe), and outcomes reported in various continuous outcome scales. For the primary outcome, we will consider the outcomes reported closest to 4 weeks after the study interventions. Pain relief measured at this time will give us indication of treatment efficacy beyond the effects of placebo. For the primary analysis, we will use a complete case analysis with ITT. Analysis and synthesis will be done using revman (review manager) 5.3. Using random effects model for pooling, we will calculate the risk ratio (to be interpreted as the risk of having success) for dichotomous outcomes and weighted mean difference (WMD) for continuous outcomes converted into 0–10 (11 point) numerical rating scale (NRS) for pain. Further, we will dichotomize all included patients into proportion of successful patients for pain relief, to be able to report risk ratio across all included studies. However, dichotomising continuous outcomes will result in a loss of statistical power. Hence, we will also report the WMD. To achieve this, we will be performing the following methodological steps.

1. *Converting outcomes reported in various continuous scales to a reference instrument scale of 0 to 10 NRS:* According to IMMPACT, the 11-point NRS measure of pain intensity is recommended as a core outcome measure in clinical trials of chronic pain treatments. Furthermore, the 0–10 NRS is preferred over the VAS (visual analog scale) by patients and clinicians for its relative simplicity and ease of administration [58].
2. *Converting continuous outcomes from 0–10 NRS into dichotomous data*. A reduction of two points on the 0–10 NRS could be considered as the threshold to establish minimally important difference (MID), reflecting an average reduction of pain by 30 % [58, 62]. For studies, which report only end scores, we will consider a threshold of 4 or less in a 0–10 NRS, to dichotomize the patients. A score of 4 or less is considered as mild pain [63].
3. *Converting categorical measurement results to a dichotomous data*. The categories of ordinal scale commonly used in pain studies could include mild, moderate and severe pain. It has been shown that these categories correspond to the following thresholds of 0–10 NRS. Mild pain 0–4, moderate pain 5–7, and severe pain 8–10 [63]. Any change from a higher ordinal category to a lower ordinal category would be considered as a successful outcome for patients reported in a particular study, allowing us to dichotomize the study outcomes into success and failures for each arm.
4. *Imputation for participants treated as lost to follow up for continuous and dichotomous outcomes (for sensitivity analyses)*. We will consider patient LTFU subsequent to randomization as missing for data analysis, and will be explored further for imputation, if it is >5 %. For trials in which the authors report total missing participant data only, without specifying at what stage the participants were missing, we will consider the total sample size and the actual sample size included for final analysis and assume that missing data were equally distributed between the arms. For trials in which the authors reported imputed analysis only, we will use the imputed results for the meta-analysis. We will perform imputational strategies as described by Ebrahim et al. [64] and Akl et al. [65], for continuous measures and dichotomous measures, respectively.

For secondary outcomes, we will pool the outcomes for other IMMPACT domains using a random effects model only if there are three or more studies for a particular domain. We will report the pooled outcome as WMD or standardized mean difference (SMD) as appropriate.

### Risk of bias assessment and identification

Each included study will be assessed for risk of bias at the study level, using the Cochrane risk of bias tool, based on the components of random sequence generation, allocation concealment, blinding of participants, blinding of outcome assessment, incomplete outcome data, and selective outcome reporting. We will use a modified Cochrane risk of bias instrument, with response options of “definitely yes,” “probably yes,” “probably no,” and “definitely no.” We will assign trials in the “definitely yes” and “probably yes” categories a high risk of bias and those in the “probably no” and “definitely no” categories a low risk of bias. These items for each study will be extracted, in duplicate, during the data extraction stage, using the DistillerSR web tool. Any disagreement on the risk of bias item scoring will be noted and arbitrated by the primary investigator (HS). Agreement on risk of bias scoring will be assessed on a component-by-component basis, using quadratic kappa weighting and interpreted as stated before. No study will be excluded based on its risk of bias. We will contact study authors if limitations in reporting lead to uncertainties in eligibility, risk of bias, or outcome.

### Assessment of heterogeneity

Statistical heterogeneity will be calculated using Cochrane’s Q test, with a threshold of *p* value at 0.10, and *I*^2^ statistic to describe the percentage variability in individual effect estimates that could be due to true differences between the studies rather than a sampling error.

### Subgroup and sensitivity analysis to explore heterogeneity

The following a priori hypotheses would be considered for subgroup analyses, and as possible reasons for unexplained heterogeneity in the pooled estimates. The subgroup analysis will only be done if there are more than two studies per subgroup.

1. *Clinical category level subgroup analysis*. Although, the underlying rationale that adding steroids to LA may not provide any meaningful improvement to pain reduction is consistent across all clinical indications and trials, there is potential for clinical heterogeneity in these conditions because of the variation in underlying pain pathways, pain sensitization, and hence the possibility of varying effects of steroids. Considering this, we will perform subgroup analysis of pain outcomes based on target structures injected. The seven clinical categories considered are as follows.
  1. Peripheral joint: hip, knee, shoulder joint injections, and other joint injections.
  2. Spinal joint/facet nerve (medial branch block)/ spinal disc: Facet joint or disc injections.
  3. Spinal nerve or epidural or intrathecal: Epidural injections including transforaminal epidural.
  4. Peripheral nerve: various nerves including occipital nerve, suprascapular, and median nerve.
  5. Autonomic ganglia: injections to stellate ganglia, celiac plexus, lumbar sympathetic block.
  6. Soft tissue injection: injections for lateral or medial elbow ligaments, subacromial bursa, or plantar fascia.
  7. Trigger point or intramuscular: injections to trigger point, piriformis muscle, or any other intramuscular injections.

2. *Larger effects toward steroids could be observed in studies with components of higher risk of bias*; we will conduct this subgroup analysis on a risk of bias component-by-component basis, only if there is considerable variability within the risk of bias component.
3. *Sensitivity analysis for LTFU will be conducted as described previously.*
4. *Studies which involved treatment interventions as a series, rather one specific injection* (for example, three steroid injections over 1 month). We anticipate that the direction of effect would favor the steroid treatment due to systemic additive effect.

### Addressing potential biases

Publication bias will be assessed using a funnel plot, if there are more than ten studies included in a meta-analysis. Further, it will be explored by the test of Egger [66]. Selective outcome reporting is difficult to identify when the study protocols are not available or published. For our review, we will consider the possibility of selective outcome reporting, when the outcomes are described in the Methods section but not identified or reported in the results section of the same study report [67].

### Interpretation and reporting

We will report our findings as risk ratios, WMD, or SMD for individual outcomes, along with their 95 % confidence intervals. Within each clinical category (subgroups), similar findings will be reported. We will also report the outcome for pain relief as a single pooled estimate of risk ratio by dichotomising the continuous outcomes. This method has been suggested as appropriate and meaningful for interpretation in systematic reviews of pain studies [54]. We will also report the findings in measures of relative risk reduction and absolute risk reduction. Rating of quality of evidence will also be done using the Grading of Recommendations Assessment, Development, and Evaluation (GRADE) approach. This will enable us to report our study findings in the form of “summary of findings” table and allow us to evaluate the certainty in effect estimates.

## Discussion

Despite the several advances in the field of chronic pain, we still do not fully understand the nature and mechanisms causing CNCP. Although steroids are being used in nearly every injection or interventional therapy, the reasons for its use are unclear. Our comprehensive systematic review will assess the clinical benefits of steroids when used along with LA, by comparing it with the effects of LA alone. This evaluation of both the effectiveness and the adverse events will include patient-important outcomes as outlined in IMMPACT recommendations, thereby enhancing comparability and external validity. Our results will facilitate evidence-based management of patients with chronic non-cancer pain and identify key areas for future research.

The review as such will be limited by the included studies and their quality. As highlighted above, we will attempt to decrease the potential for bias by performing reasonable subgroup and sensitivity analysis, including imputation for missed outcomes.

## Additional file

Additional file 1:
**Search strategy.**

## Abbreviations

CLBP: chronic low back pain
CNCP: chronic non-cancer pain
CSI: corticosteroid injections
ESI: epidural steroid injections
IA: intra-articular injection
IMMPACT: Initiative on Methods, Measurement, and Pain Assessment in Clinical Trials
ITT: intention to treat
LA: local anesthetics
LE: lateral epicondylitis
LTFU: loss to follow up
ME: medial epicondylitis
MID: minimally important difference
NRS: numerical rating scale
OS: observational study
PNB: peripheral nerve block
RCT: randomized controlled trials
RD: risk difference
SMD: standardized mean difference
TFESI: transforaminal epidural steroid injection
TPI: trigger point injection
VAS: visual analog scale
WMD: weighted mean difference
