# Supplementary material for: Local anesthetic injections with or without steroid for chronic non-cancer pain: a protocol for a systematic review and meta-analysis of randomized controlled trials
Source: Syst Rev. 2016 Feb 1;5:18. doi: 10.1186/s13643-016-0190-z (PMC4736179; doi:10.1186/s13643-016-0190-z)
Supplement: Additional file 1: — Search strategy. [file 13643_2016_190_MOESM1_ESM.doc]

**Appendix 1: Search Strategy**

MEDLINE, April 20, 2015

Database: Ovid MEDLINE(R) In-Process & Other Non-Indexed Citations, Ovid MEDLINE(R) Daily and Ovid MEDLINE(R) <1946 to Present>

Search Strategy:

--------------------------------------------------------------------------------

1 exp Anesthetics, Local/ (91356)

2 (lidocaine or lignocaine or bupivicaine or mepivacaine or ropivacaine or levobupivicaine or prilocaine).mp. [mp=title, abstract, original title, name of substance word, subject heading word, keyword heading word, protocol supplementary concept word, rare disease supplementary concept word, unique identifier] (33604)

3 1 or 2 (98302)

4 exp Injections/ (247829)

5 injection*.mp. (610031)

6 4 or 5 (620262)

7 exp Nerve Block/ (16723)

8 (nerve adj block).mp. [mp=title, abstract, original title, name of substance word, subject heading word, keyword heading word, protocol supplementary concept word, rare disease supplementary concept word, unique identifier] (18379)

9 7 or 8 (18395)

10 6 or 9 (634595)

11 exp Steroids/ (712950)

12 exp adrenal cortex hormones/ or exp glucocorticoids/ (342120)

13 (steroid* or corticosteroid* or hydrocortisone or glucocorticoid* or cortisol or cortisone).tw. (350443)

14 (dexamethasone or depomedrol or methylprednisolone or prednisone or triamcinolone or betamethasone).mp. (136627)

15 or/11-14 (939357)

16 6 and 15 (61311)

17 9 and 15 (879)

18 16 or 17 (61812)

19 10 and 15 (61812)

20 3 and 19 (2002)

21 randomized controlled trial.pt. (392167)

22 controlled clinical trial.pt. (89258)

23 randomized.ab. (316812)

24 placebo.ab. (161159)

25 drug therapy.fs. (1762677)

26 randomly.ab. (228616)

27 trial.ab. (327431)

28 groups.ab. (1439740)

29 or/21-28 (3506766)

30 exp animals/ not humans.sh. (4025936)

31 20 and 29 (1332)

32 limit 20 to "therapy (best balance of sensitivity and specificity)" (502)

33 randomized controlled trial.pt. or randomized.mp. or placebo.mp. (650209)

34 20 and 33 (502)

35 31 or 32 (1335)

36 35 not 30 (1203)

EMBASE, April 21, 2015

Database: Embase <1974 to 2015 April 20>

Search Strategy:

1 exp local anesthetic agent/ (194103)

2 (lidocaine or lignocaine or bupivicaine or mepivacaine or ropivacaine or levobupivicaine or prilocaine).mp. [mp=title, abstract, subject headings, heading word, drug trade name, original title, device manufacturer, drug manufacturer, device trade name, keyword] (75448)

3 1 or 2 (196901)

4 exp injection/ (107360)

5 #.mp. (611695)

6 4 or 5 (618075)

7 exp nerve block/ (27338)

8 (nerve adj block).mp. [mp=title, abstract, subject headings, heading word, drug trade name, original title, device manufacturer, drug manufacturer, device trade name, keyword] (24578)

9 7 or 8 (29406)

10 6 or 9 (641978)

11 exp steroid/ (1219649)

12 exp glucocorticoid/ or exp corticosteroid/ (756096)

13 (steroid* or corticosteroid* or hydrocortisone or glucocorticoid* or cortisol or cortisone).tw. (448844)

14 (dexamethasone or depomedrol or methylprednisolone or prednisone or triamcinolone or betamethasone).mp. [mp=title, abstract, subject headings, heading word, drug trade name, original title, device manufacturer, drug manufacturer, device trade name, keyword] (342874)

15 or/11-14 (1331227)

16 6 and 15 (64860)

17 9 and 15 (2702)

18 16 or 17 (66784)

19 10 and 15 (66784)

20 3 and 19 (5346)

21 clinical article/ (1478872)

22 exp clinical study/ (6955375)

23 clinical trial/ (846792)

24 controlled study/ (4572842)

25 randomized controlled trial/ (369824)

26 major clinical study/ (2325582)

27 double blind procedure/ (121982)

28 multicenter study/ (119299)

29 single blind procedure/ (19967)

30 phase 3 clinical trial/ (20059)

31 phase 4 clinical trial/ (1769)

32 crossover procedure/ (42310)

33 placebo/ (266847)

34 or/21-33 (9803613)

35 allocat$.mp. (104792)

36 assign$.mp. (261613)

37 blind$.mp. (348841)

38 (clinic$ adj25 (study or trial)).mp. (4194615)

39 compar$.mp. (5258635)

40 control$.mp. (7094611)

41 cross?over.mp. (69730)

42 factorial$.mp. (50497)

43 follow?up.mp. (914570)

44 placebo$.mp. (348754)

45 prospectiv$.mp. (714094)

46 random$.mp. (1118421)

47 ((singl$ or doubl$ or trebl$ or tripl$) adj25 (blind$ or mask$)).mp. (227037)

48 trial.mp. (1366112)

49 (versus or vs).mp. (1299041)

50 or/35-49 (12425407)

51 34 and 50 (7585297)

52 exp animals/ or exp invertebrate/ or animal experiment/ or animal model/ or animal tissue/ or animal cell/ or nonhuman/ (21443714)

53 human/ or normal human/ or human cell/ (15844186)

54 52 and 53 (15797455)

55 52 not 54 (5646259)

56 51 not 55 (5833541)

57 20 and 56 (2644)

58 random:.tw. (971230)

59 placebo:.mp. (348754)

60 double-blind:.tw. (155665)

61 58 or 59 or 60 (1191245)

62 20 and 61 (1282)

63 56 and 62 (1142)

64 57 or 63 (2644)

CENTRAL, April 21, 2015

ID Search Hits

#1 MeSH descriptor: [Anesthetics, Local] explode all trees 6037

#2 local anesthetic:ti,ab,kw (Word variations have been searched) 8549

#3 lidocaine or lignocaine or bupivicaine or mepivacaine or ropivacaine or levobupivicaine or prilocaine:ti,ab,kw (Word variations have been searched) 9901

#4 #1 or #2 or #3 13628

#5 MeSH descriptor: [Injections] explode all trees 18650

#6 injection:ti,ab,kw (Word variations have been searched) 45184

#7 #5 or #6 45222

#8 MeSH descriptor: [Nerve Block] explode all trees 2680

#9 nerve block:ti,ab,kw (Word variations have been searched) 4849

#10 #8 or #9 4852

#11 #7 or #10 48484

#12 MeSH descriptor: [Steroids] explode all trees 38474

#13 MeSH descriptor: [Adrenal Cortex Hormones] explode all trees 11466

#14 MeSH descriptor: [Glucocorticoids] explode all trees 3499

#15 steroid* or corticosteroid* or hydrocortisone or glucocorticoid* or cortisol or cortisone:ti,ab,kw (Word variations have been searched) 35431

#16 dexamethasone or depomedrol or methylprednisolone or prednisone or triamcinolone or betamethasone:ti,ab,kw (Word variations have been searched) 15413

#17 #12 or #13 or #13 or #14 or #15 or #16 65973

#18 #7 and #17 6064

#19 #10 and #17 476

#20 #18 or #19 6365

#21 #11 and #17 6365

#22 #4 and #21 in Trials 669

**WHO ICTRP (**June 1, 2015)

Title: "local anesthetic" or steroid

AND

Intervention: injection

AND Recruitment status is: ALL

**ClinicalTrials.gov (**June 1, 2015)

Search Terms: local anesthetic or steroid

AND

Intervention: injection
